# Bovine Herpesvirus 1 Productive Infection Led to Inactivation of Nrf2 Signaling through Diverse Approaches

**DOI:** 10.1155/2019/4957878

**Published:** 2019-09-15

**Authors:** Xiaotian Fu, Dongmei Chen, Yan Ma, Weifeng Yuan, Liqian Zhu

**Affiliations:** ^1^College of Veterinary Medicine, Yangzhou University and Jiangsu Co-Innovation Center for Prevention and Control of Important Animal Infectious Diseases and Zoonoses, Yangzhou 225009, China; ^2^Laboratory Animal Center, Chinese Center for Disease Control and Prevention, Beijing 102206, China; ^3^Keck School of Medicine, University of Southern California, Los Angeles, CA 90033, USA; ^4^Institute of Animal Sciences, Chinese Academy of Agricultural Sciences, Beijing 100193, China

## Abstract

Bovine herpesvirus type 1 (BoHV-1) is a significant cofactor for bovine respiratory disease complex (BRDC), the most important inflammatory disease in cattle. BoHV-1 infection in cell cultures induces overproduction of pathogenic reactive oxygen species (ROS) and the depletion of nuclear factor erythroid 2 p45-related factor 2 (Nrf2), a master transcriptional factor regulating a panel of antioxidant and cellular defense genes in response to oxidative stress. In this study, we reported that the virus productive infection in MDBK cells at the later stage significantly decreased the expression levels of heme oxygenase-1 (HO-1) and NAD(P)H quinone oxidoreductase-1 (NQO1) proteins, the canonical downstream targets regulated by Nrf2, inhibited Nrf2 acetylation, reduced the accumulation of Nrf2 proteins in the nucleus, and relocalized nuclear Nrf2 proteins to form dot-like staining patterns in confocal microscope assay. The differential expression of Kelch-like ECH associated protein 1 (KEAP1) and DJ-1 proteins as well as the decreased association between KEAP1 and DJ-1 promoted Nrf2 degradation through the ubiquitin proteasome pathway. These data indicated that the BoHV-1 infection may significantly suppress the Nrf2 signaling pathway. Moreover, we found that there was an association between Nrf2 and LaminA/C, H3K9ac, and H3K18ac, and the binding ratios were altered following the virus infection. Taken together, for the first time, we provided evidence showing that BoHV-1 infection inhibited the Nrf2 signaling pathway by complicated mechanisms including promoting Nrf2 degradation, relocalization of nuclear Nrf2, and inhibition of Nrf2 acetylation.

## 1. Introduction

Bovine herpesvirus type 1 (BoHV-1) belongs to the family *Herpesviridae* and the subfamily *Alphaherpesvirinae*. As an important pathogen for cattle of all ages and breeds, the virus infection causes diverse clinical symptoms, such as inflammation in the upper respiratory tract and reproductive system [1]. The erosion of mucosal surfaces and disruption of immune protection attributed to the virus infection may lead to a secondary infection, which consequently leads to a disease known as bovine respiratory disease complex (BRDC), the most important disease in cattle [2, 3]. Therefore, BoHV-1 is regarded as a critical cofactor for BRDC development. The virus infection as well as the virus-induced BRDC causes great economic loss to the cattle industry worldwide [3].

Under physiological conditions, the production of reactive oxidative species (ROS) [4], such as hydrogen peroxide (H_2_O_2_), peroxynitrite (OONO^−^), and hydroxyl radicals (OH^·^), is tightly controlled by both intracellular enzymatic and nonenzymatic antioxidants responsible for ROS neutralization and removal. Therefore, ROS are generated and maintained at relatively low levels and act as second messengers to regulate diverse biological processes, such as gene expression, cell proliferation, and differentiation [5–8]. However, the overproduction of ROS induced by various factors, including virus infection, can result in pathogenic oxidative stress [9, 10]. Accumulating studies have indicated that excessive ROS play important roles in the pathogenesis of inflammatory diseases. For example, ROS contribute to virus replication and the activation of inflammatory cytokines during the infection of both influenza virus and HSV-1 [6, 7, 11, 12]; thereby, the inhibition of ROS production is regarded as a potential therapeutic approach against virus infection [13]. The overproduction of ROS in BoHV-1-infected cells has been demonstrated to enhance viral replication, mitochondrial dysfunction, and DNA damage [4, 14, 15]. It is well established that the redox-sensitive transcription factor, NF-E2-related factor 2 (Nrf2), and Nrf2 antioxidant response pathways provide the primary cellular defenses against the oxidative stress [16, 17]. We have recently reported that BoHV-1 infection in MDBK cells inhibited Nrf2 expression [18].

Under normal conditions, Nrf2 is constitutively expressed and remains inactive in the cytosol by binding to its inhibitor protein Kelch-like ECH-associated protein 1 (KEAP1) to form a complex, which promotes the degradation of Nrf2 via the ubiquitin proteasome pathway [19]. In response to oxidative or electrophilic stress, Nrf2 is released from the KEAP1 complex and translocates to the nucleus, where it forms a heterodimer with small Maf (musculoaponeurotic fibrosarcoma) (sMaf) protein and binds to the DNA at the site of the antioxidant response element (ARE) [20, 21]. ARE sequences are conserved regions in the promotors of antioxidant and detoxifying genes, such as heme oxygenase-1 (HO-1), NAD(P)H quinone oxidoreductase-1 (NQO1), glutamate cysteine ligase catalytic and regulatory subunits (GCLC and GCLM), glutathione S-transferase (GST), uridine diphosphate glucuronosyltransferase (UDPGT), superoxide dismutase (SOD), catalase (CAT), glucose 6 phosphate dehydrogenase (G6PD), and glutathione peroxidase-1 (GPx) [22–24]. Recently, multiple studies have shown that the Nrf2 signaling pathway can either be stimulated or be inactivated by diverse viruses, contributing to viral pathogenesis and disease progression [23, 25]. For example, Nrf2 signaling is stimulated by the infection of influenza virus, but is suppressed during RSV infection, and the Nrf2-deficient mice exhibit much stronger inflammatory responses following either virus infection [26–29]. It is evident that intact Nrf2 signaling is essential for protecting a host against virus infection, and the dysfunction of Nrf2 signaling is potentially involved in viral pathogenesis.

Though we have recently demonstrated that BoHV-1 infection destabilized Nrf2 protein expression [18], the status of Nrf2-regulated antioxidant signaling and the mechanisms underlying Nrf2 depletion in the virus-infected cells have yet to be determined. Here, for the first time, we demonstrated that the virus infection suppressed the Nrf2 signaling pathway through multiple approaches, including inhibiting of the Nrf2 accumulation in the nucleus, inducing the relocalization of nucleus Nrf2 to form dot-like structures and the deacetylation of nucleus Nrf2, and manipulating the DJ-1/KEAP1 complex to promote the degradation of Nrf2 via the ubiquitin proteasome pathway.

## 2. Materials and Methods

### 2.1. Cells and Virus

MDBK cells were cultured in DMEM (Gibco BRL) containing 10% horse serum (HyClone Laboratories, Logan, UT, USA). BoHV-1 (Colorado1 stain) was propagated in MDBK cells. Aliquots of the virus stocks were tittered in MDBK cells and stored at −80°C.

### 2.2. Antibodies and Chemicals

The following chemicals were used in this study: tert-Butyl hydroperoxide solution (tBHP) (Sigma, cat# 458139), MG132 (Sigma, cat# 474791-1), and Trolox (MedChemExpress, cat# HY-101445). The following antibodies were used in this study: Nrf2 rabbit polyclonal antibody (Abcam, cat# ab137550), LaminA/C mouse monoclonal antibody (Santa Cruz Biotechnology, cat# sc-376248), phospho-(Ser/Thr) Phe rabbit polyclonal antibody (Cell Signaling Technology, cat# 9631), HO-1 mouse monoclonal antibody (Enzo Life Sciences, cat# ADI-OSA-110-D), NQO1 rabbit polyclonal antibody (ABclonal, cat# A0047), DJ-1 (ABclonal, cat# A0201), KEAP1 (D6B12) rabbit monoclonal antibody (Cell Signaling Technology, cat# 8047), acetyl-Histone H3 (Lys9) rabbit monoclonal antibody (Cell Signaling Technology, cat# 9649), acetyl-Histone H3 (Lys18) rabbit monoclonal antibody (Cell Signaling Technology, cat# 13998), ubiquitin mouse monoclonal antibody (Cell Signaling Technology, cat# 3936), pan acetyl Lysine rabbit polyclonal antibody (ABclonal, cat# A2391), *β*-actin rabbit monoclonal antibody (Cell Signaling Technology, cat# 4970), Mouse Control IgG (ABclonal, cat# AC011), rabbit control IgG (ABclonal, cat# AC005), BoHV-1 VP16 rabbit polyclonal antibody (kindly provided by Prof. Vikram Misra at the University of Saskatchewan [30]), HRP- (horseradish peroxidase-) conjugated goat anti-mouse IgG (Cell Signaling Technology, cat# 7076), and goat anti-rabbit IgG (Cell Signaling Technology, cat# 7074).

### 2.3. Western Blot Analysis

MDBK cells were seeded into 60 mm dishes and cultured overnight and subsequently were infected with BoHV-1 (MOI = 0.1) for 4, 8, 16, 24, 36, and 48 hours. Cell lysates were prepared using lysis buffer (1% Triton X-100, 50 mM sodium chloride, 1 mM EDTA, 1 mM EGTA, 20 mM sodium fluoride, 20 mM sodium pyrophosphate, 1 mM phenylmethylsulfonyl fluoride, 0.5 g/mL leupeptin, 1 mM benzamidine, and 1 mM sodium orthovanadate in 20 mM Tris-HCl, pH 8.0). After centrifugation at 13,000 rpm for 10 min at 4°C, the clarified supernatants were collected and boiled together with Laemmli sample buffer for 10 min, and the samples were then separated on 8% or 10% SDS-PAGE and proteins were transferred onto PVDF membranes (Bio-Rad, cat# 1620177). After blocking with 5% nonfat milk in PBS (pH, 6.8) for 1 hour (h) at room temperature, the membranes were incubated with primary antibodies diluted in 5% bovine serum albumin in PBS (pH, 6.8), overnight at 4°C. All the primary antibodies were diluted 1 : 1000 for Western blots unless indicated specifically. After extensive washing with PBST (0.1% Tween 20 in PBS, pH 6.8), the membranes were incubated with secondary antibodies (1 : 1000) of either anti-rabbit or anti-mouse for 1 hour at room temperature. After extensive washing with PBST, the protein bands were developed using Clarity Western ECL Substrate (Bio-Rad, cat# 1705061). The band intensity was analyzed using software ImageJ.

To analyze the effects of chemicals on the expression of indicated proteins, MDBK cells in 60 mm dishes were pretreated with either solvent DMSO or the individual chemicals at indicated concentrations for 1 h; then the cells were infected with the virus (MOI = 0.1) for 24 h in the presence of the chemicals or DMSO solvent. The cell lysates prepared using the lysis buffer as described above were subjected to Western blot analysis.

### 2.4. Immunoprecipitation (IP) Assay

MDBK cells grown in 60 mm dishes were mock infected or infected with BoHV-1 (MOI = 0.1). At 24 h after infection, cells were lysed with 600 *μ*L of RIPA buffer (1x PBS, 1% NP-40, 0.5% sodium deoxycholate, 0.1% SDS) supplemented with aforementioned protease inhibitors. The cell lysates were then clarified by centrifugation at 13,000 rpm for 10 minutes and incubated with Dynabeads protein A (Life Technologies, cat# 10001D), which have been precoated with primary antibodies by incubation for 1 h at room temperature with rotation. After overnight incubation at 4°C with rotation, the beads were collected with the help of a magnet (DynaMag™) (Life Technologies, cat# 12321D). After three washings with PBS (pH 6.8), the beads were boiled in SDS-loading buffer and Western blots were performed using the indicated antibodies.

### 2.5. Immunofluorescence Assay (IFA)

MDBK cells seeded into 2-well chamber slides (Nunc Inc., IL, USA) were mock infected or infected with BoHV-1 (MOI = 0.1) for 24 h. Cells were fixed with 4% paraformaldehyde prepared in PBS (pH 7.4) for 10 min at room temperature, permeabilized with 0.25% Triton X-100 in PBS (pH 7.4) for 10 min at room temperature, and blocked with 1% BSA in PBST (PBS+ 0.1% Tween 20) for 1 h followed by the incubation with the antibody against Nrf2 (1 : 500 dilution) and/or LaminA/C (1 : 500) in 1% BSA in PBST for 12 h at 4°C. After three washings, the cells were incubated with Alexa Fluor 488®-conjugated goat anti-rabbit IgG (H+L) (Invitrogen, cat# A-11008, 1 : 500 dilution) and/or Alexa Fluor 633®-conjugated goat anti-mouse IgG (H+L) (Invitrogen, cat# A-21052, 1 : 500 dilution) for 1 h in the dark. After three washings, DAPI (4′,6-diamidino-2-phenylindole) staining was performed to visualize nuclei. Slides were covered with coverslips by using of antifade mounting medium (Electron Microscopy Sciences, cat# 50-247-04). Images were captured by using a confocal microscope (Leica).

### 2.6. Relative Quantification of mRNA by qRT-PCR

Total RNA was extracted from infected cells or uninfected control cells using a TRIzol LS reagent (Ambion, cat# 10296010) following the manufacturer's instructions. Freshly prepared total RNA (1 *μ*g) was used as a template for the synthesis of first-strand cDNA with commercial random hexamer primers using Thermoscript™ RT-PCR system Kit (Invitrogen, cat# 11146-024) following the manufacturer's instructions. The cDNA products were then used as templates for real-time quantitative PCR to measure mRNA levels of indicated genes with gene-specific primers. For these studies, we analyzed HO-1 (forward primer: 5′-CAGAAGATGTAGCCAGAGCA-3′ and reverse primer: 5′-CATAGGGCAAGCGGTCA-3′) [31], NQO1 (forward primer: 5′-TGTATGCCATGAACTTCAATCC-3′ and reverse primer: 5′-AGTCTCGGCAGGATACTGAAAG-3′) [31], Nrf2 (forward primer: 5′-TCCAGGCGGATTCTTTACCA-3′ and reverse primer: 5′-TGACGCACCTCCCATTTCTC-3′) [32], and GAPDH (forward primer: 5′-CCATGGAGAAGGCTGGGG-3′ and reverse primer: 5′-AAGTTGTCATGGATGACC-3′) [25].

Analysis of glyceraldehyde-3-phosphate dehydrogenase (GAPDH) mRNA was used as an internal control. Real-time PCR was carried out using the ABI 7500 fast real-time system (Applied Biosystems, CA). The expression levels of the tested genes were normalized to that of the GAPDH gene. The relative mRNA level of each gene was calculated using the method (2^-*ΔΔ*CT^) by a comparison to the control.

## 3. Results

### 3.1. BoHV-1 Infection Suppressed Nrf2 Signaling

**Figure 1:**
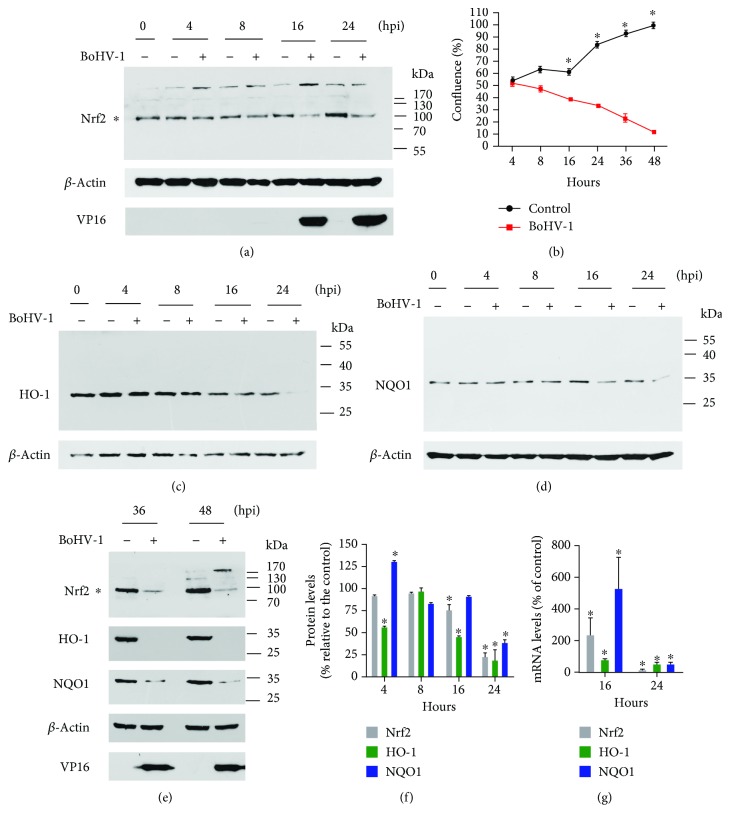
The effects of BoHV-1 infection on the expression of Nrf2-regulated downstream targets. (a, c, d, and e) MDBK cells in 60 mm dishes were mock infected or infected with BoHV-1 at an MOI of 0.1 for 4, 8, 16, 24, 36, and 48 h. The cell lysates were then prepared for Western blots to detect the expression of Nrf2, HO-1, NQO1, and VP16 using the Nrf2 antibody (Abcam, cat# ab137550, 1 : 500), HO-1 monoclonal antibody (mouse) (Enzo Life Sciences, cat# ADI-OSA-110-D, 1 : 1000), NQO1 polyclonal antibody (ABclonal, cat# A0047, 1 : 1000), and VP16 antibody (a gift from Prof. Vikram Misra at the University of Saskatchewan, 1 : 2000). (b) MDBK cells were seeded into 24-well plates. After overnight incubation, the cells were infected with BoHV-1 at an MOI of 0.1. After infection for 4, 8, 16, 24, 36, and 48 h, the cells were collected and cell numbers were counted using a Trypan-blue exclusion test. Data shown are representative of three independent experiments. (f) The band intensity was analyzed with software ImageJ. Each analysis was compared with that of uninfected control at each time point, which was arbitrarily set as 100%. These images are representative of those from three independent experiments. (g) Total RNA was prepared at 16 and 24 hpi in MDBK cells, and the mRNA levels of Nrf2, HO-1, and NQO1 were measured by qRT-PCR. Each analysis was compared with that of uninfected control, which was arbitrarily set as 100%. Data represent three independent experiments. The significance was assessed with Student's *t*-test (^∗^*P* < 0.05).

We have recently reported that the steady-state protein levels of Nrf2 are significantly reduced during BoHV-1 productive infection at the later stage [18]; however, whether the Nrf2 signaling pathway is consequently inhibited remains unknown. In order to evaluate the activity of Nrf2 signaling, we first detected the protein levels of HO-1 and NQO1, the canonical downstream targets regulated by Nrf2 signaling. Our data indicated that the virus infection altered the protein expression of Nrf2, HO-1, and NQO1 with diverse kinetics prior to 16 h after infection (Figures 1(a), 1(c), 1(d), and 1(f)). But from 24 to 48 hours post infection (hpi), the protein levels of all three molecules were consistently reduced (Figures 1(a) and 1(c)–1(f)). Particularly at 36 and 48 hpi, HO-1 protein expression was reduced to a level barely detectable. Quantitative analysis indicated that at 24 hpi the expression levels of Nrf2, HO-1, and NQO1 proteins were reduced to 23.2%, 19.3%, and 39.2% relative to the control, respectively (Figure 1(f)). To validate the correlation between virus infection and these varied protein expressions, we performed cell proliferation assay following virus infection and detected the protein expression of viral protein VP16. As shown in Figure 1(b), the cell proliferation was significantly inhibited from 16 hpi and gradually decreased until 48 hpi, which correlated with the positive detection of viral protein VP16 from 16 to 48 hpi (Figures 1(a) and 1(e)). Therefore, the virus-productive infection kinetics supported the finding that the virus infection modulated the protein expression of Nrf2, HO-1, and NQO1. In addition, we found that at 24 hpi, but not at 16 hpi, the mRNA levels of Nrf2 and NQO1 were consistent with those at the protein level (Figures 1(f) and 1(g)). Since HO-1 and NQO1 proteins are widely used indicators of Nrf2 activity, these data suggest that BoHV-1 infection at a later stage suppresses the Nrf2 signaling pathway.

**Figure 2:**
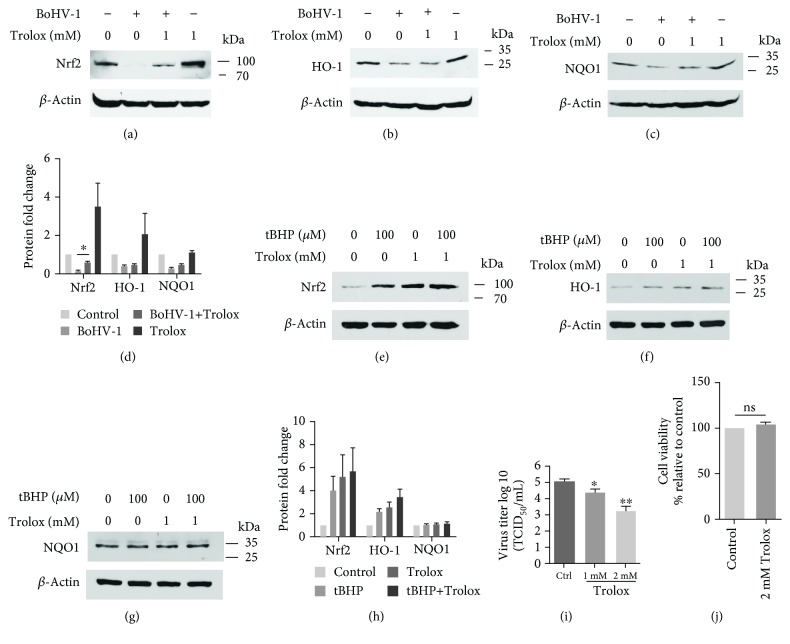
The effects of Trolox on the expression of Nrf2 and its downstream targets. (a, b, and c) MDBK cells in 60 mm dishes pretreated with Trolox (1 mM) or DMSO control for 1 h were infected with BoHV-1 (MOI = 0.1); in the presence of Trolox or DMSO control for 24 h, the cell lysates were prepared for Western blots to detect the expression of Nrf2 (a), HO-1 (b), and NQO1 (c). (e, f, and g) MDBK cells in 60 mm dishes pretreated with Trolox (1 mM) or DMSO control for 1 h were exposed to tBHP in the presence of Trolox or DMSO control for 2 h; the cell lysates were prepared for Western blots to detect the expression of Nrf2 (e), HO-1 (f), and NQO1 (g). (d and h) The relative band intensity was analyzed with software ImageJ, and each analysis was compared with that of uninfected control at each time point, which was arbitrarily set as 100%. Data shown are representative of three independent experiments. (i) MDBK cells in 24-well plates pretreated with Trolox at indicated concentrations or MDSO control were infected with BoHV-1 (MOI = 0.1) for 24 hours in the presence of an inhibitor or DMSO. The cell cultures were subjected to frozen-thawing twice, and viral yield was determined with the results being expressed as TCID_50_/mL. (j) The cytotoxicity of Trolox in MDBK cells for 24 h was analyzed by Trypan-blue exclusion. The significance was assessed with Student's *t*-test (^∗^*P* < 0.05).

Mechanistically, the Nrf2 signaling can be affected by either a ROS-dependent or ROS-independent pathway [33, 34]. Trolox, a chemical having strong capacity to neutralize intracellular ROS with minor off-target effects, has been widely used to study the interaction between ROS and Nrf2 signaling; e.g., it has been reported that Trolox contributes to Nrf2-mediated protection from injury by cigarette smoke in human and murine primary alveolar cells [35]. As expected, Trolox at a concentration of 1 and 2 mM showed no cytotoxicity to MDBK cells, but significantly reduced the virus production in a dose-dependent manner (Figures 2(i) and 2(j)). The treatment of virus-infected cells with 1 mM Trolox could partially restore Nrf2 depletion induced by virus infection albeit not to the initial level (Figures 2(a) and 2(d)). However, the Trolox treatment could not partially restore the depletion of either HO-1 or NQO1 protein at 24 hpi (Figures 2(b)–2(d)). We concluded that ROS may play a minor role in regulating Nrf2 signaling transduction during virus infection.

To confirm that the Trolox used in this study functioned properly, the effect of hydrogen peroxide (tBHP) on the activation of Nrf2 in the presence/absence of Trolox was examined. We found that either tBHP or Trolox along could stimulate the expression of both Nrf2 and HO-1 proteins, and the stimulatory effects of tBHP were further boosted by Trolox. However, neither of them had effects on the NQO1 protein expression in MDBK cells (Figures 2(e)–2(h)). These data confirmed that Trolox used in this study functioned as predicted, which validated its effects on the Nrf2 expression during virus infection.

### 3.2. BoHV-1 Infection Stimulated Nrf2 Degradation through the Ubiquitin Proteasome Pathway

**Figure 3:**
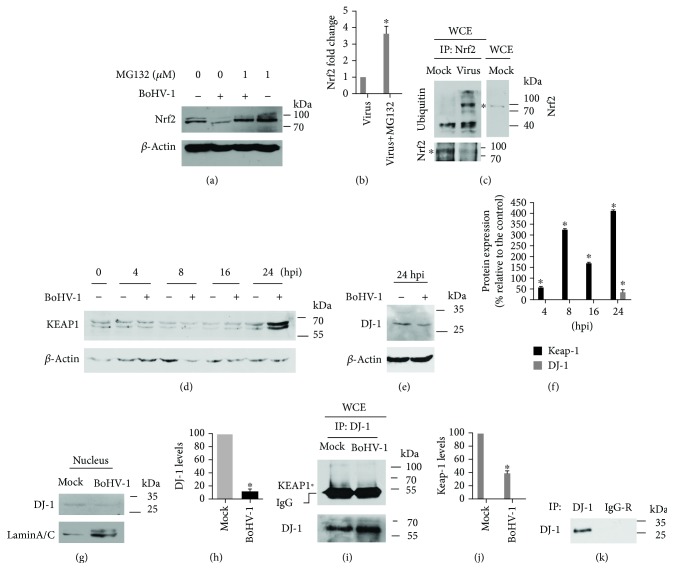
BoHV-1 infection promoted Nrf2 depletion through a proteasome pathway. (a, c, and i) MDBK cells in 60 mm dishes were infected with BoHV-1 at an MOI of 0.1 in the presence of MG132 (1 *μ*M) or DMSO control. After infection for 24 h, the cell lysates were prepared for Western blotting to detect the expression of Nrf2 (a) or were prepared for IP using antibodies against Nrf2 (c), DJ-1 (d), and unrelated IgG (k). The IP samples were subjected to immunoblots using antibodies against ubiquitin (Cell Signaling Technology, cat# 3936, 1 : 1000) and Nrf2 (Abcam, cat# ab137550, 1 : 500), respectively. (d and e) MDBK cells in 60 mm dishes were mock infected or infected with BoHV-1 (MOI = 0.1) for an indicated time length. The cell lysates were then prepared for Western blots to detect the expression of KEAP1 and DJ-1 using antibodies against KEAP1 mAb (Cell Signaling Technology, cat# 8047, 1 : 1000) and DJ-1 (ABclonal, cat# A0201, 1 : 1000), respectively. Data shown are representative of three independent experiments. (f, h, and j) The band intensity was analyzed with software ImageJ. Each analysis was compared with that of control, which was arbitrarily set as 100%. These images are representative of those from three independent experiments. The significance was assessed with Student's *t*-test (^∗^*P* < 0.05). (g) MDBK cells in 60 mm dishes were mock infected or infected with BoHV-1 (MOI = 0.1) for 24 hpi. Then, the nuclear proteins were prepared using a commercial kit, and the levels of protein DJ-1 were detected by immunoblots. Data shown are representative of three independent experiments. WCE: whole cell extract.

It has been reported that the degradation of Nrf2 protein through the ubiquitin proteasome pathway is regulated by either KEAP1-dependent or KEAP1-independent mechanisms [36]. To confirm that the ubiquitin proteasome pathway was involved in Nrf2 degradation in the virus-infected MDBK cells, the cells were exposed to proteasome inhibitor MG132 throughout viral infection as described previously [37]. We have reported that MG132 at a concentration of 1 *μ*M had no cytotoxicity to MDBK cells [37]. The treatment of MG132 (1 *μ*M) partially restored the Nrf2 depletion induced by the virus infection, and its protein level was increased to ~3.5-fold relative to the mock-treated infected control (Figures 3(a) and 3(b)), indicating that the ubiquitin proteasome pathway was potentially involved in the Nrf2 depletion in virus-infected cells. To provide direct evidence that Nrf2 was ubiquitinated, the lysates of either virus-infected or mock-infected cells were subjected to immunoprecipitation (IP) assay using the antibody against Nrf2; subsequently, the extents of ubiquitination of the Nrf2 protein were detected by immunoblots using the antibody against ubiquitin. To accurately determine the identity of the Nrf2 bands, both IP samples and whole cell extract (WCE) were loaded in the same gel spaced by a lane loaded by a protein ladder marker. The membrane was cut into two pieces along with the protein marker, and both ubiquitin and Nrf2 were incubated with an individual primary antibody, respectively. They were finally combined together and developed at the same time in parallel. So the bands with molecular weights showing the same size as that in Nrf2 were regarded as the ubiquitinated Nrf2. As expected, the high levels of ubiquitin were detected in virus infected cell cultures (Figure 3(c)), which further confirmed that virus infection enhanced the ubiquitination levels of Nrf2. Collectively, these results suggested that the ubiquitin proteasome pathway was involved in the degradation of Nrf2 following virus infection.

KEAP1 is known to mediate the degradation of Nrf2 protein through the ubiquitin proteasome pathway. We then examined the effects of virus infection had on the expression of KEAP1 in MDBK cells. As shown in Figures 3(d) and 3(f), the protein levels of KEAP1 were consistently increased from 8 to 24 hpi. Particularly, it was increased approximately 4-fold at 24 hpi compared to the control. So the increased expression of KEAP1 protein may potentially contribute to Nrf2 degradation through the ubiquitin proteasome pathway.

DJ-1 can stabilize Nrf2 expression through preventing protein association between Nrf2 and KEAP1 [38, 39]. Our study demonstrated that the expression of DJ-1 protein was significantly reduced at 24 hpi (Figures 3(e) and 3(f)). Moreover, we found that virus infection destabilized the protein association between DJ-1 and KEAP1 (Figures 3(i) and 3(j)), which further supported the findings that virus infection promoted the ubiquitination of Nrf2 (Figure 3(c)). In addition, specific binding to DJ-1 by the antibody used in IP was confirmed (Figure 3(k)). It is reasonable to speculate that the reduced expression levels of DJ-1 protein result in the release of more KEAP1 from the KEAP1-DJ-1 complex, which consequently sequesters more cytosol Nrf2 and ultimately promotes Nrf2 degradation through the ubiquitin proteasome pathway. Taken together, these data implied that BoHV-1 infection promoted Nrf2 degradation partially through the regulation of the KEAP1 and DJ-1 complex.

In addition, the accumulation of DJ-1 protein in the nucleus was also inhibited at 24 hpi (Figure 3(g)). Quantitative analysis indicated that the protein levels of DJ-1 in the nucleus were decreased to ~12.15% in the virus-infected cells (at 24 hpi) relative to the mock-infected control (Figure 3(h)). It has been previously reported that nuclear translocation of DJ-1 protects neurons from cell death after oxidative stress [40]. Therefore, we speculated that the reduced accumulation of DJ-1 protein in the nucleus following virus infection is a potential pathogenic effect of the virus infection.

### 3.3. Virus Infection Relocalized Nuclear Nrf2 and Inhibited the Accumulation of Nrf2 Protein in the Nucleus

**Figure 4:**
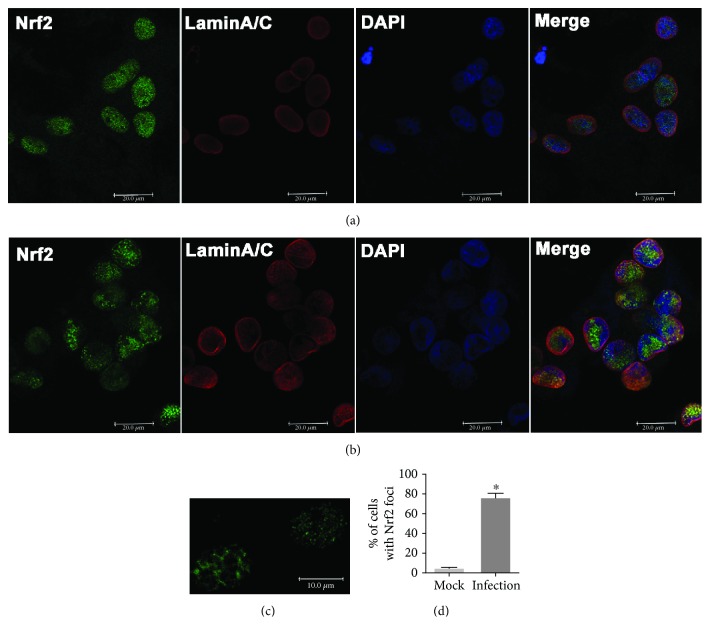
BoHV-1 infection altered the localization of nuclear Nrf2 protein. MDBK cells in 2-well chamber slides were mock infected (a) or infected with BoHV-1 (MOI = 0.1) (b) for 24 hours. After three washings with PBS, cells were fixed with 4% formaldehyde, and Nrf2 was detected by IFA using the Nrf2 antibody (1 : 500) and LaminA/C antibody (1 : 500). DAPI staining was used to stain nuclear DNA. Images were obtained by performing confocal microscopy (Leica). These images are representative of three independent experiments. (c) Zoom-in cells showing typical dot-like staining. (d) The percentage of dot-like staining-positive cells among ~400 cells was estimated from photos derived from three independent experiments. Scale bar: 200 *μ*M.

**Figure 5:**
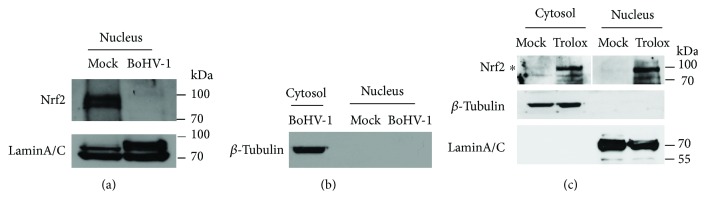
BoHV-1 infection altered the accumulation of Nrf2 in the nucleus. (a) MDBK cells in 60 mm dishes were mock infected or infected with BoHV-1 (MOI = 0.1) for 24 h, the cell cultures were collected for extracting nuclear proteins using commercial nuclear protein purification kit (Beyotime Biotechnology, cat# P0027). Nrf2 was detected by Western blot using the antibody against Nrf2 (Abcam, cat# ab137550). LaminA/C was detected and used as protein loading control. (b) Protein fractions of both the cytosol and nucleus were subjected to Western blots using the antibody against *β*-tubulin. (c) MDBK cells in 60 mm dishes were mock treated or treated with Trolox (1 mM) for 2 h; the cell lysates were collected for the purification of nuclear proteins and cytosol proteins using commercial nuclear protein purification kit (Beyotime Biotechnology, cat# P0027). Nrf2 was detected by Western blot using the antibody against Nrf2 (Abcam, cat# ab137550). LaminA/C and *β*-tubulin were detected to characterize whether each fraction was contaminated. These images are representative of three independent experiments.

To understand whether BoHV-1 infection influences the subcellular localization of Nrf2, an immunofluorescence assay (IFA) was performed at 24 hpi. Interestingly, in mock-infected MDBK cells, Nrf2 was predominantly detected in the nucleus but not in the cytosol (Figure 4(a)). Similarly, in the virus-infected cells, Nrf2 was mainly present in the nucleus rather than in the cytosol, either (Figure 4(b)). These findings were not in line with the canonical model that Nrf2 is mainly inactivated and sequestered by KEAP1 in the cytosol, in unstressed conditions, and once stimulated, it is translocated into the nucleus [36]. Of note, the virus infection destabilized Nrf2 protein steady-state expression (Figure 1(a)), so in the confocal microscopy, longer exposure time was required to detect the presence of Nrf2 protein in the virus-infected cells relative to that in the uninfected control; thus, one cannot compare Nrf2 levels between these IFA images. Our results suggested that in MDBK cells Nrf2 was mainly located in the nucleus. To examine whether Nrf2 protein in MDBK cells is active prior to viral infection, the cells were treated with Trolox; both cytoplasmic and nuclear proteins were extracted for the detection of Nrf2 proteins. As shown in Figure 5(c), the expressions of Nrf2 protein from both cytosol and nuclear fractions were significantly increased in comparison to those in the mock-treated controls (Figure 5(c)), suggesting that Nrf2 was readily activated by Trolox in cell culture. Thus, these data indicated that MDBK cells used in this study were not in an activated status, which validated our novel findings that Nrf2 was mainly distributed in the nucleus rather than in the cytosol in either mock or infection contexts.

Interestingly, immunofluorescence revealed a dot-like staining pattern for nuclear Nrf2 in the virus-infected cells (Figures 4(b) and 4(c)). Following viral infection, approximately 74.5% of the cells exhibited a dot-like staining pattern in the nucleus while in mock infection control only ~4.1% of the cells show faint dot-like staining (Figure 4(d)), suggesting that virus infection led to the relocalization of Nrf2 in the nucleus.

Nuclear translocation of Nrf2 protein is essential for subsequent transactivation of its downstream target genes. Therefore, the accumulation of Nrf2 in the nucleus is a canonical indicator of Nrf2 activation. To test whether BoHV-1 productive infection altered Nrf2 nuclear accumulation, MDBK cells were mock infected or infected for 24 h; nuclear proteins were purified by using a commercial kit. As a result, there was a significant decrease of the nuclear Nrf2 protein level in the virus-infected cells, relative to that in mock-infected control (Figure 5(a)). A further study confirmed that the nucleus protein extraction was not contaminated by cytosol protein (Figure 5(b)), which validated the finding that the virus infection inhibited the accumulation of Nrf2 protein in the nucleus.

### 3.4. BoHV-1 Infection Inhibited the Acetylation of Nuclear Nrf2 Protein

**Figure 6:**
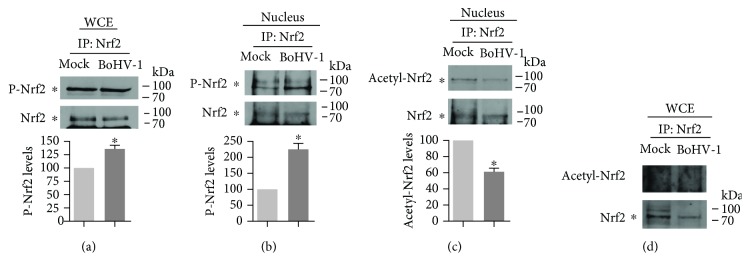
The posttranslational modification of Nrf2 stimulated by virus infection. MDBK cells in 60 mm dishes were mock infected or infected with BoHV-1 (MOI = 0.1) for 24 h; the cell cultures were collected to produce whole cell lysates (a and d) or to extract nuclear proteins using commercial nuclear protein purification kit (Beyotime Biotechnology, cat# P0027) (b and c). IP was performed using the antibody against Nrf2 (Abcam, cat# ab137550). Then, levels of phospho-Nrf2 in whole cell extracts (WCE) (a) and the nucleus (b) and acetylation levels of Nrf2 in the nucleus (c) and WCE (d) were detected by immunoblots using the antibody against the Nrf2 antibody (Abcam, cat# ab1375501:500), acetyl Lysine antibody (ABclonal, cat# A2391, 1 : 1000), and phospho-(Ser/Thr) Phe antibody (Cell Signaling Technology, cat# 9631, 1 : 1000). Data shown are representative of three independent experiments. The significance was assessed with Student's *t*-test (^∗^*P* < 0.05).

Posttranslational modification, such as by acetylation and phosphorylation, is known to regulate Nrf2 transcriptional activity [41, 42]. It has been documented that the phosphorylation and acetylation of Nrf2 are critical for the nuclear translocation and transcriptional activation [43, 44]. Therefore, the effects of virus infection on the phosphorylation and acetylation of Nrf2 were further investigated. For this purpose, Nrf2 was precipitated from either nuclear protein extract or whole cell extract, and the levels of either phosphorylated or acetylated Nrf2 protein were detected by Western blot using antibodies against phospho-(Ser/Thr) Phe and acetyl Lysine, respectively. As shown in Figures 6(a) and 6(b), the expression levels of phosphorylated Nrf2 from either whole cell or nuclear extract were increased upon virus infection. Compared to the mock-infected control, the level of phosphorylated Nrf2 protein from whole cell and nuclear extract was increased to 136.1% and 223.7% at 24 hpi, respectively (Figures 6(a) and 6(b)). Therefore, the virus infection enhanced the phosphorylation of Nrf2 proteins, particularly of those located in the nucleus.

Virus infection at 24 hpi significantly decreased the acetylation levels of the nucleus Nrf2 protein, which was reduced to ~60.7% relative to that in the mock-infected control (Figure 6(c)), which is in consistent with our findings that virus infection inhibited the accumulation of Nrf2 in the nucleus (Figure 5), and depressed the expression of HO-1 and NQO1 (Figures 1(c), 1(d), and 1(f)). Interestingly, the acetylated Nrf2 from the whole cell extract was not readily detected in either mock-infected or virus-infected cell cultures (Figure 6(d)). We speculated that the acetylated Nrf2 protein mainly accumulates in the nucleus because the acetylation of Nrf2 increases its transcriptional activity and nucleus localization, whereas the deacetylation leads to Nrf2 nuclear export [42]. Taken together, BoHV-1 infection inhibited Nrf2 acetylation, further supporting our hypothesis that BoHV-1 infection inactivates the Nrf2 signaling pathway.

### 3.5. BoHV-1 Infection Altered the Association Ratio between Nrf2 and LaminA/C

**Figure 7:**
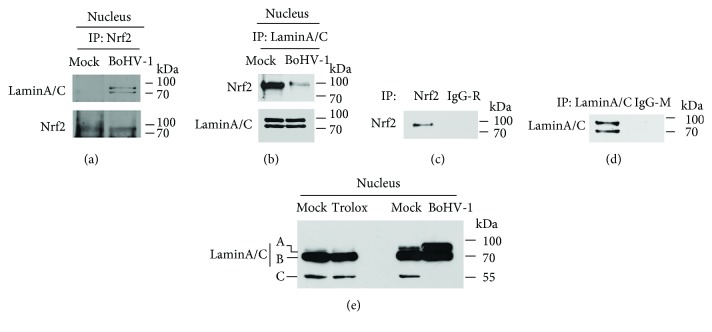
BoHV-1 affected the association between Nrf2 and LaminA/C. MDBK cells in 60 mm dishes were mock infected or infected with BoHV-1 for 24 h. Nucleus proteins were purified using commercial nuclear protein purification kit (Beyotime Biotechnology, cat# P0027). IP was performed using antibodies against Nrf2 (a), LaminA/C (b), Nrf2 and unrelated IgG (c), and LaminA/C and IgG (d) (R: rabbit; M: mouse). Then, Western blots were performed using corresponding antibodies. (e) MDBK cells in 60 mm dishes were mock treated or treated with Trolox (1 mM) for 2 h. Nucleus proteins were purified using commercial nuclear protein purification kit (Beyotime Biotechnology, cat# P0027), and LaminA/C was detected by Western blots.

To better understand how the virus infection relocalizes Nrf2, the antibody against LaminA/C was used for specific staining of the nuclear membrane. We found that nuclear Nrf2 proteins partially colocalized with LaminA/C in the virus-infected cells (Figure 4(b)). We speculated that Nrf2 was associated with LaminA/C. To address this issue, nuclear proteins were extracted from either mock-infected or virus-infected MDBK cells, which were then subjected to IP assay using antibodies against either Nrf2 or LaminA/C. As a result, when the antibody against Nrf2 was used in the IP, much more LaminA/C was precipitated in the virus-infected cells (Figure 7(a)). While when IP was performed using the antibody against LaminA/C, less Nrf2 was precipitated in the virus-infected cells (Figure 7(b)). Based on these results, we assumed that if the ratio between Nrf2 and LaminA/C was arbitrarily set as 1 (Nrf2 : LaminA/C = 1) in the control, the ratio would be less than 1 (Nrf2 : LaminA/C < 1) in the virus-infected cells, suggesting that the virus infection reduced the association ratio between Nrf2 and LaminA/C. Unrelated IgG was included to do IP, which confirmed that both antibodies can be used for IP and validated that the target proteins can be specifically precipitated (Figures 7(c) and 7(d)). This was an interesting finding which needs extensive study to reveal the biological functions for this association in the future.

The LaminA/C antibody recognizes two bands with molecular weights ranging from 65 to 70 kDa. In this study, we found that this monoclonal antibody recognized three bands which were denoted by A, B, and C in Figure 7(e), when the samples from either mock treated or treated with Trolox were applied for Western blots. However, when the samples from the virus-infected cells were applied for Western blots, band C disappeared, but the intensity of band A increased, which was largely different from that in the control. This finding indicated that BoHV-1 infection altered the LaminA/C expression pattern. Since LaminA/C is associated with Nrf2, it was reasonable to speculate that the altered expression manners of LaminA/C would affect Nrf2 relocalization in the nucleus.

### 3.6. BoHV-1 Infection Altered the Association Ratio between Nrf2 and H3K9ac/H3K18ac

**Figure 8:**
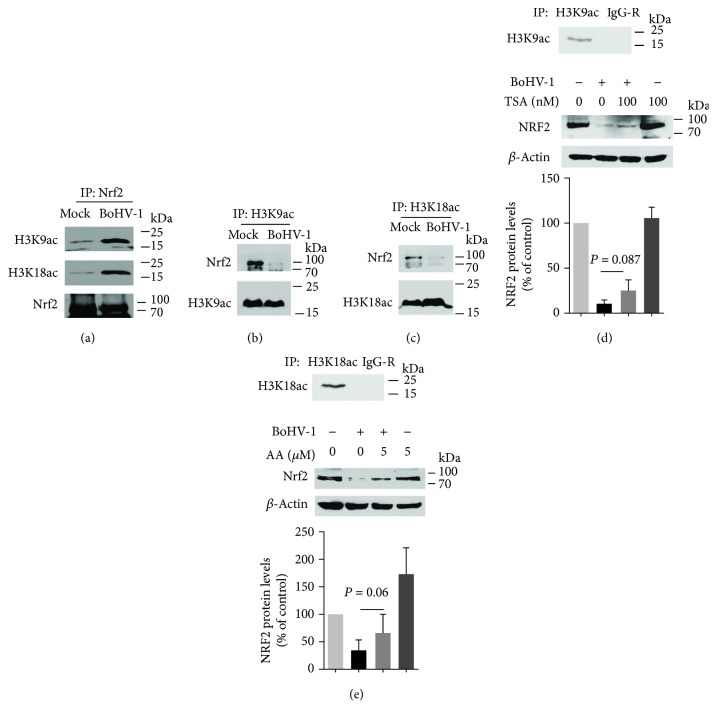
BoHV-1 affected the association between Nrf2 and H3K9ac/H3K18ac. MDBK cells in 60 mm dishes were mock infected or infected for 24 h. The cell lysates were prepared to perform IP using antibodies against Nrf2 (a), H3K9ac (b), H3K18ac (c), H3K9ac and rabbit IgG (d), and H3K18ac and rabbit IgG (e). The precipitated proteins were subjected to Western blots using the following antibodies: Nrf2 (Abcam, cat# ab137550, 1 : 1000), acetyl-Histone H3 (Lys9) rabbit mAb (Cell Signaling Technology, cat# 9649, 1 : 1000), and acetyl-Histone H3 (Lys18) rabbit mAb (Cell Signaling Technology, cat# 13998, 1 : 1000). Data shown are representative of three independent experiments. The significance was assessed with Student's *t*-test (^∗^*P* < 0.05).

In eukaryotes, DNA is packaged into a protein-DNA complex called chromatin. Histone H3, an important component of chromatin, can regulate the activity of chromatin through diverse epigenetic modifications, such as acetylation and phosphorylation. The acetylation of certain lysine (K) residues such as K9 and K18 in histone H3 is an indicator of transcriptionally active chromatin [45]. Whether histones, such as H3K9ac and H3K18ac, are associated with Nrf2 is currently known. To further understand how the virus infection relocalizes nuclear Nrf2, the association between Nrf2 and the acetylated H3 including H3K9ac and H3K18ac was detected. For this purpose, IP was performed using antibodies against Nrf2, H3K9ac, or H3K18ac, respectively (Figures 8(a)–8(c)). Also, unrelated IgG was included to confirm that these antibodies worked well for IP and validate that the target proteins were precipitated specifically (Figures 8(d) and 8(e)). We demonstrated that Nrf2 stably associated with both H3K9ac and H3K18ac in both mock-infected and virus-infected MDBK cells (Figures 8(a)–8(c)). When the antibody against Nrf2 was used to do IP, much more H3K9ac and H3K18ac were precipitated in the virus-infected cells (Figure 8(a)).When IP was performed using antibodies against either H3K9ac or H3K18ac, less Nrf2 was precipitated in the virus-infected cells (Figure 8(b)). Based on these results, we assumed that if the ratio between Nrf2 and H3K9ac/H3K18ac was arbitrarily set as 1 (Nrf2 : H3K9ac/H3K18ac = 1) in the control, the ratio would be less than 1 (Nrf2 : H3K9ac/H3K18ac < 1) in the virus-infected cells, suggesting that the virus infection reduced the association ratio between Nrf2 and H3K9ac/H3K18ac. Since we have previously reported that the virus infection significantly reduced the protein levels of both H3K9ac and H3K18ac [37], the association between Nrf2 and H3K9ac/H3K18ac supported our findings that virus infection relocalized nuclear Nrf2. It is highly possible that more Nrf2 protein was relocalized to the transcriptionally active chromatin in the virus-infected cells, which is a possible mechanism to overcome adverse effects of Nrf2 depletion during virus infection.

## 4. Discussion

The transcriptional activator Nrf2 plays a vital role in maintaining cellular homeostasis through regulating the expression of multiple antioxidant proteins, detoxification enzymes, and xenobiotic transporters [46, 47]. Nrf2 also regulates multiple cellular processes including cell differentiation, proliferation, and inflammation [36], which may be closely related to virus pathogenesis. BoHV-1 infection significantly reduces Nrf2 expression which is inconsistent with the report that virus infection enhances ROS production in cell cultures [4, 15, 18]. Here, we raised questions of whether and how the virus infection inhibits the Nrf2 signaling pathway.

In this study, we provided evidence that (i) BoHV-1 infection (after 24 hpi) significantly reduced the expression of HO-1 and NQO-1 (Figure 1), the known downstream proteins regulated by Nrf2 signaling, (ii) virus infection significantly reduced accumulation of Nrf2 in the nucleus (Figure 5), (iii) virus infection induced the deacetylation of nucleus Nrf2 (Figure 6), and (iv) virus infection led to relocalization of nuclear Nrf2 and forming a dot-like structure (Figure 4). These data unanimously suggested that BoHV-1 infection at a later stage led to the suppression of the Nrf2 signaling pathway.

HO-1 and NQO-1 are known downstream proteins regulated by Nrf2 signaling. In this study, we found that the treatment with Trolox could partially restore Nrf2 depletion induced by virus infection albeit not to the initial level, but it had no effects on the protein levels of either HO-1 or NQO-1 (Figure 2). These data implied that the virus infection hijacked the Nrf2 signaling pathway and preferentially controlled these Nrf2 downstream targets using Nrf2-independent manners.

In a canonical theory, Nrf2 is sequestered in the cytosol by forming a complex with KEAP1 which targets Nrf2 for proteasome degradation. Once activated, the complex is destructed, and therefore, Nrf2 is released and enters the nucleus where it stimulates the expression of downstream target genes. However, our data indicated that in MDBK cells either with or without infection, Nrf2 protein was readily detected in the nucleus rather than in the cytosol (Figures 4(a) and 4(b)). This is a novel finding which is in contrast to the canonical model that Nrf2 translocates to the nucleus under stimulation. We speculated that the cellular localization of Nrf2 protein is cell-type specific.

Of note, nuclear Nrf2 was relocalized following virus infection, forming dot-like structures identified by IFA (Figure 4(b)). Interestingly, partial part of Nrf2 colocalized with LaminA/C, and the association between LaminA/C and Nrf2 was confirmed by IP assay using the antibody against either Nrf2 or LaminA/C (Figure 7). Considering that the virus infection altered the expression patterns of LaminA/C and reduced the association ratio between Nrf2 and LaminA/C (Figure 7), it is reasonable to speculate that these changes may affect relocalization of nuclear Nrf2.

By using ChIP assay, it has been revealed that both phosphorylated histone H3 at serine 10 (H3S10) and Nrf2 are recruited to the promoter region of HO-1, and consequently, they coordinately stimulate HO-1 transcription in response to arsenite stimulation [48], suggesting a relationship between H3S10 and Nrf2. Here, we provided evidence showing that Nrf2 associated with activated histone H3 such as H3K9ac and H3K18ac (Figure 8). Since H3K9ac and H3K18ac are markers of transcriptional active chromatin, it is highly possible that Nrf2 preferentially binds to transcriptional active chromatin to overcome the adverse effects of Nrf2 depletion following virus infection. In addition, the virus infection reduced the association ratio between Nrf2 and H3K9ac/H3K18ac (Figure 8) and decreased the protein levels of both H3K9ac and H3K18ac [37], which potentially induce nuclear Nrf2 relocalization. This is an interesting finding which needs further study to reveal the biological effects of these associations.

Phosphorylation of Nrf2 by multiple kinases, such as PKC [44], AMPK [49], and CK2 [43], is critical for nuclear translocation and transcription activation. In this study, we found that the total phosphorylation levels of nuclear Nrf2 protein were significantly increased following virus infection (Figure 6). Of note, in this study, the phospho-(Ser/Thr) Phe antibody recognizing pan-phosphorylation sites was applied; therefore, the data represented the total phosphorylation status of Nrf2 proteins. The phosphorylation status in certain residues of Nrf2 such as Ser558 or serine 40 is critical for Nrf2 nuclear accumulation [49–51]. While the relationship between the total phosphorylation status of Nrf2 and Nrf2 activity has not been reported, our results provided a possibility that the increasing levels of phosphorylated Nrf2 proteins are negatively related to Nrf2 nuclear accumulation.

Diverse cellular factors, such as KEAP1 and DJ-1, may potentially influence Nrf2 stabilization and activation. Nrf2 expression can be regulated with KEAP1-dependent and KEAP1-independent mechanisms [36]. In this study, our findings indicated that BoHV-1 infection promoted the proteasome degradation of Nrf2 (Figure 2(a)) in line with the increased expression of KEAP1 protein following the virus infection (Figure 2(d)), suggesting that KEAP1 is potentially involved in the virus infection-induced degradation of Nrf2. DJ-1 may potentially stabilize Nrf2 expression through competitively binding to KEAP1 protein [38, 52, 53]. It has been reported that the activation of the DJ-1/Nrf2 pathway is involved in the pathogenesis of diabetic nephropathy in rats [54]. In this study, our findings indicated that BoHV-1 infection decreased DJ-1 expression, which may result in a decreased capability of binding to Nrf2 through competing with KEAP1. Immunoprecipitation using the antibody against DJ-1 further confirmed that virus infection led to a reduced capacity of binding to KEAP1. Therefore, virus infection rendered DJ-1 loss capacity to protect Nrf2 degradation which facilitated the degradation of Nrf2 through the ubiquitin proteasome pathway mediated by KEAP1.

Taken together, in this study, we provided evidences indicating that BoHV-1 infection led to an inactivation of the Nrf2 signaling pathway through complicated mechanisms such as by relocalization of nuclear Nrf2, deacetylation of nuclear Nrf2, and inhibition of Nrf2 nucleus translocation, as well as by promoting Nrf2 degradation through lost capacity of DJ-1. This study would extend our understanding of the mechanism of the virus infection-induced oxidative stress.

## Data Availability

The data used to support the findings of this study are available from the corresponding author upon request.
